# Chidamide combined with a modified Bu-Cy conditioning regimen improves survival in patients with T-cell acute lymphoblastic leukemia/lymphoma undergoing allogeneic hematopoietic stem cell transplantation

**DOI:** 10.1007/s00277-024-05849-y

**Published:** 2024-06-20

**Authors:** Xuanqi Cao, Zheng Li, Yanming Zhang, Qingya Cui, Haiping Dai, Yunju Ma, Mengyun Li, Sifan Chen, Jia Yin, Wei Cui, Jia Chen, Aining Sun, Huiying Qiu, Suning Chen, Xiaming Zhu, Borje S. Andersson, Depei Wu, Xiaowen Tang

**Affiliations:** 1 National Clinical Research Center for Hematologic Diseases, Jiangsu Institute of Hematology, The First Affiliated Hospital of Soochow University, Suzhou, China; 2 Institute of Blood and Marrow Transplantation, Collaborative Innovation Center of Hematology, Soochow University, Suzhou, China; 3 Department of Hematology, Affiliated Huai’an Hospital of Xuzhou Medical University, Huai’an, China; 4 Department of Stem Cell Transplantation and Cellular Therapy, The University of Texas M.D. Anderson Cancer Center, Houston, TX USA

**Keywords:** Allogeneic stem cell transplantation, Conditioning regimen, T acute lymphoblastic leukemia/Lymphoma, Histone deacetylase inhibitors

## Abstract

This study aimed to evaluate the efficacy and safety of chidamide (Chi) combined with a modified Busulfan-Cyclophosphamide (mBuCy) conditioning regimen for T-cell acute lymphoblastic leukemia/lymphoma (T-ALL/LBL) patients undergoing allogeneic hematopoietic stem cell transplantation (allo-HSCT). Twenty-two patients received chidamide combined with mBuCy conditioning regimen (Chi group). A matched-pair control (CON) group of 44 patients (matched 1:2) received mBuCy only in the same period. The leukemia-free survival (LFS), overall survival (OS), cumulative incidence of relapse (CIR), and non-relapse-related mortality (NRM) were evaluated. Patients in the Chi group were associated with lower 2-year CIR (19.0 vs. 41.4%, *P* = 0.030), better 2-year LFS (76.1 vs. 48.1%, *P* = 0.014), and had no significant difference in 2-year OS (80.5 vs. 66.4%, *P* = 0.088). Patients with minimal residual disease (MRD) positive before HSCT in the Chi group exhibited an advantage in 2-year LFS and a trend towards better 2-year OS (75.0 vs. 10.2%, *P* = 0.048; 75.0 vs. 11.4%, *P* = 0.060, respectively). Multivariable analysis showed that the chidamide intensified regimen was independently associated with better LFS (HR 0.23; 95%CI, 0.08–0.63; *P* = 0.004), and showed no significant impact with OS for all patients (HR 0.34, 95%CI, 0.11–1.07; *P* = 0.064). The cumulative incidence rates of grade II-IV aGVHD were similar (36.4 vs. 38.6%, *P* = 0.858). 20 patients in Chi group evinced an elevation in γ-glutamyltransferase, as compared to the mBuCy group (90.9 vs. 65.9%, *P* = 0.029). No transplantation-related mortality was documented within the first 100 days after transplantation. The results demonstrate that the chidamide intensified regimen may be an effective and acceptable safety option for T-ALL/LBL undergoing allo-HSCT, and further validation is needed.

**Supplementary Information:**

The online version contains supplementary material available at 10.1007/s00277-024-05849-y.

## Introduction

T-cell acute lymphoblastic leukemia/lymphoma (T-ALL/LBL) has a poor response to chemotherapy and an extremely bad prognosis due to its tendency to high white blood cells, central nervous system, and extramedullary infiltration [1–5]. Allogeneic hematopoietic stem cell transplantation (allo-HSCT) is a potentially curative treatment [6, 7]. However, the cumulative incidence of post-transplant relapse in T-ALL/LBL was 30 to 50%, constituting a major cause of patient mortality [8–12]. And the 2-year overall survival (OS) probability after relapse was about 10 to 46% [9, 12, 13]. Intensifying conditioning regimens is one of the potential ways to reduce relapse rates. The modified Bu-Cy (mBuCy) conditioning regimen is the predominant myeloablative therapy in China, owing to its low transplant-related mortality and efficient hematopoietic reconstitution [14, 15]. However, when administered to T-ALL/LBL patients, this regimen remains limited by its inability to eliminate minimal residual disease (MRD) and high incidence of relapse following transplantation. Therefore, further refinement and optimization of the T-ALL/LBL conditioning regimen is imperative.

As the first oral subtype-selective histone deacetylase inhibitor (HDACi) approved in China [16], the real-world study has demonstrated that chidamide has a favorable efficacy and an acceptable safety profile for T-lymphocytic malignancies patients [17]. Chidamide could inhibit proliferation and induce apoptosis via cell cycle arrest and the regulation of apoptotic proteins in T-cell malignancies [18, 19]. Of note, chidamide combined with chemotherapy regimens can decrease MRD in T-ALL patients with *NOTCH1* mutation [20]. Given its promising antitumor activity and synergistic effects, Ji et al. incorporated chidamide into the lymphoma pretreatment regimen (ChiCGB). The 4-year progression-free survival (PFS) and OS rates for high-risk or relapsed/refractory lymphomas were significantly improved, reaching 80.6% and 86.1%, respectively [21]. To date, the role of chidamide in T-ALL/LBL conditioning regimens in allo-HSCT has not been explored. Here, we retrospectively compared the clinical efficacy and safety between mBuCy combined with chidamide and mBuCy conditioning regimen for T-ALL/LBL patients undergoing allo-HSCT.

## Methods

### Patients

We conducted a retrospective study at the First Affiliated Hospital of Soochow University between December 21, 2017, and February 21, 2022, to evaluate the safety and efficacy of the chidamide-containing conditioning regimen for high-risk T-ALL/LBL. 22 patients received the chidamide combined with modified Bu-Cy regimen (Chi group), 44 similar patients received only modified Bu-Cy (CON group) and underwent allo-HSCT during the same period. Disease types included T-ALL/LBL, early T-cell precursor acute lymphoblastic leukemia (ETP-ALL) and T-lymphoid/myeloid mixed phenotype acute leukemia (T/M MPAL). This study was approved by the ethics committee of the First Affiliated Hospital of Soochow University, and all patients provided written informed consent before treatment in accordance with the Helsinki Declaration.

### Propensity score matching

To reduce the impact of potential confounders, we employed propensity score matching (PSM). The following matching variables were used as covariates: age and sex of patients, diagnosis, disease status before transplantation, donor/recipient gender match, donor type, source of stem cells and whether patients were relapsed or refractory. Optimal pair matching based on propensity scores in a 1:2 ratio was conducted, with a caliper of 0.2 standard deviations. A propensity score test validated the normalization impact of covariate matching, as indicated in Table S1.

### Conditioning regimens

Patients in the Chi group received chidamide in combination with the mBuCy as a conditioning regimen. If the donors were haploidentical type, chidamide was administered 15 mg orally on days − 14, -12, -9, -7, and − 5 before chemotherapy. The patients received Me-CCNU 250 mg/m^2^/d orally on day − 12, cytarabine 2 g/m^2^ i.v. every 12 h on days − 11 to − 10 for a total of 4 doses, busulfan 0.8 mg/kg i.v. every 6 h on days − 9 to − 7 (12 doses), and cyclophosphamide 1.8 g/m^2^/d i.v. on days − 5 and − 4. If the donors were HLA-matched siblings or unrelated types, chidamide was administered 15 mg orally on days − 11, -9, -7, and − 5 before chemotherapy. The patients received Me-CCNU 250 mg/m^2^/d orally on day − 11, cytarabine 2 g/m^2^ i.v. every 12 h on day − 10, busulfan 0.8 mg/kg i.v. every 6 h on days − 9 to − 7 (12 doses), and cyclophosphamide 1.8 g/m^2^/d i.v. on days − 5 and − 4. Patients in the CON group only received the same mBuCy regimen except for chidamide.

### Graft-versus-host disease prophylaxis

The graft-versus-host disease (GVHD) prophylaxis protocol is the same as our center’s previously published study [22, 23]. The prophylaxis regimen included cyclosporine A (CsA) with short-course methotrexate (MTX) for related identical donor transplantation, and CsA, short-course MTX, mycophenolate mofetil (MMF), and rabbit anti-thymocyte globulin (Thymoglobulin™) for unrelated and haploidentical transplantation. CsA 3 mg/kg/d was continuously infused for 24 h from day − 9, of which the trough concentration was adjusted to 150–200 ng/ml. It was switched to oral administration when the patient’s bowel function normalized again after the transplant. From day − 9, 0.5 g of MMF was administered orally every 12 h and was discontinued on day + 28 post-transplant. Short-course MTX was administered on days + 1 (15 mg/m^2^), + 3, +6, and + 11 (10 mg/m^2^). Rabbit-ATG (2.5 mg/kg/d, i.v.) was used from day − 5 to − 2.

### Definitions and statistical methods

OS was the time from transplantation to death. Leukemia-free survival (LFS) was the time from transplantation to leukemia relapse, death, or last follow-up. Non-relapse-related mortality (NRM) was defined as the time from transplantation to death of any causes other than hematologic disease relapse. Relapse was defined as the reappearance of leukemia cells (≥ 5%) in the peripheral blood or bone marrow of patients in complete remission or the development of extramedullary disease. Refractory was defined as (1) Failure of initial induction therapy after two or more courses of treatment; (2) early recurrence less than 6 months after the first remission; (3) inefficacy to response to induction chemotherapy after recurrence; and (4) multiple relapses. For univariate comparison, the Mann–Whitney U test was used for continuous variables, and the chi-square test or Fisher’s exact test for categorical variables. The Cox proportional hazards model was used for univariate and multivariate analysis. OS and LFS were estimated by the Kaplan–Meier method and compared by using the log-rank test. The CIR was estimated using Gray’s sub-distribution method to account for the presence of competing risk due to non-relapse mortality. *P* values < 0.05 were considered significant. All analyses in this study were performed using the IBM Statistical Package for Social Sciences version 25 and R version 4.2.2.

## Results

### Patient enrollment and characteristics

66 patients with T-ALL/LBL were screened and enrolled, and 22 patients received a chidamide-containing conditioning regimen. Patients’ characteristics are shown in Table 1. The median age at transplantation was 28 (range, 19–37) years, and 59 (89.4%) of 66 patients were male. The median time from diagnosis to HSCT was 179 (range, 122–229) days. The pre-transplant characteristics were similar between the two groups.

**Table 1:** Transplant characteristics

Characteristic	Total	CON (*n* = 44)	Chi (*n* = 22)	*P* value
Gender, n (%)				
Male	59 (89.4%)	40 (90.9%)	19 (86.4%)	0.678
Female	7 (10.6%)	4 (9.1%)	3 (13.6%)	
Median age, years (range)	28 (19–37)	28 (19–37)	29 (20–34)	0.744
Diagnosis, n (%)				
T-ALL	33 (50.0%)	22 (50.0%)	11 (50.0%)	0.979
T-LBL	23 (34.8%)	18 (22.0)	5 (22.7%)	
ETP-ALL	9 (13.6%)	5 (11.4%)	4 (18.2)	
T/M MPAL	11 (16.7%)	9 (20.5%)	2 (9.1)	
Time from diagnosis to HSCT (d), median (range)	179 (122–229)	163 (112–217)	185 (147–267)	0.174
Status of disease before HSCT, n (%)				
CR1	51 (77.3%)	34 (77.3%)	17 (77.3%)	1.000
≥CR2	9 (13.6%)	6 (13.6%)	3 (13.6%)	
Non-CR	6 (9.1%)	4 (9.1%)	2 (9.1%)	
MRD status before HSCT, n (%)				
Negative	48 (72.7%)	30 (68.2%)	18 (81.8%)	0.241
Positive	18 (27.3%)	14 (31.8%)	4 (18.2%)	
Donor type, n (%)				
Haplo	50 (75.8%)	33 (75.0%)	17 (77.3%)	1.000
SIB	8 (12.1%)	5 (11.4%)	3 (13.6%)	
URD	8 (12.1%)	6 (13.6%)	2 (9.1%)	
Donor-recipient gender match, n (%)				
Male-male	42 (63.6%)	29 (65.9%)	13 (59.1%)	0.850
Male-female	7 (10.6%)	4 (9.1%)	3 (13.6%)	
Female-male	10 (15.2%)	7 (15.9%)	3 (13.6%)	
Female-female	7 (10.6%)	4 (9.1%)	3 (13.6%)	
Donor-recipient ABO match, n (%)				
Match	18 (27.3%)	14 (31.8%)	4 (18.2%)	0.241
Mismatch	48 (72.7%)	30 (68.2%)	18 (81.8%)	
Stem cell source, n (%)				
PBSC	54 (81.8%)	35 (79.5%)	19 (86.4%)	0.737
PBSC + BM	12 (18.2%)	9 (20.5%)	3 (13.6%)	
Number of cells infused, median (range)				
MNC (×10^8^/kg)	8.7 (7.3–11.6)	9.2 (7.6–11.8)	8.5 (6.2–10.9)	0.487
CD34+(×10^6^/kg)	3.7 (2.8-5.0)	3.5 (2.9–4.9)	3.8 (2.8–5.4)	0.876
CD3+(×10^8^/kg)	1.4 (1.2–1.9)	1.4 (1.1–1.9)	1.6 (1.3–1.8)	0.498
Poor risk factors, n (%)				
Age > 35 years at diagnosis	21 (31.8%)	16 (36.4%)	5 (22.7%)	0.262
WBC > 100 × 10^9^/L at diagnosis	14 (21.2%)	7 (15.9%)	7 (31.8%)	0.201
Extramedullary disease at diagnosis	34 (51.5%)	22 (50.0%)	12 (54.5%)	0.728
Complex karyotype	10 (15.2%)	5 (11.4%)	5 (22.7%)	0.281
Relapsed or refractory	22 (33.3%)	14 (31.8%)	8 (36.4%)	0.712

T-ALL T acute lymphoblastic leukemia, T-LBL T acute lymphoblastic lymphoma, ETP-ALL early T-cell precursor acute lymphoblastic leukemia, T/M MPAL T/myeloid mixed phenotype acute leukemia, HSCT hematopoietic stem cell transplantation, CR complete remission, MRD minimal residual disease, Haplo Haploidentical donors, SIB HLA-matched sibling donors, URD HLA-matched unrelated donors, PBSC peripheral blood stem cells, BM bone marrow, WBC white blood cell

### Engraftment and GVHD

All patients achieved hematopoietic reconstitution. The neutrophil engraftment in Chi and CON groups occurred at 11 (range, 11–13) days and 11 (range, 11–12) days (*P* = 0.412), respectively. Platelet engraftment time was 13 (range 12–17) days in the Chi group and 14 (range 12–14) days in the CON group (*P* = 0.842), respectively. The incidences of day 100 grade II–IV (36.4 vs. 38.6%; *P* = 0.858) and III–IV (13.6 vs. 20.5%; *P* = 0.735) aGVHD were not significantly different between Chi and CON groups, respectively. There was no difference in the incidence of 2-year cGVHD between the Chi group and CON group (36.4 vs. 38.5%, *P* = 0.858) (Table 2).

**Table 2:** Incidence of engraftment, GVHD, and adverse events after allo-HSCT

Engraftment, GVHD, and adverse events, *n* (%)	Chi group		CON group		*P* value
Engraftment					
Time of neutrophil reconstruction(d), median(range)	11 (11–13)		11 (11–12)		0.412
Time of platelet reconstruction(d), median(range)	13 (12–17)		14 (12–14)		0.842
GVHD					
aGVHD	11 (50.0)		25 (56.8)		0.600
grade II-IV aGVHD	8 (36.4)		17 (38.6)		0.858
grade III-IV aGVHD	3 (13.6)		9 (20.5)		0.735
cGVHD	8 (36.4)		17 (38.5)		0.858
Infection					
EBV reactivation	8 (36.4)		9 (20.5)		0.164
CMV reactivation	5 (22.7)		12 (27.3)		0.691
Pneumonia	12 (54.6)		22 (50.0)		0.728
Perianal Infection	2 (9.1)		1 (2.3)		0.531
Hemorrhagic cystitis	8 (36.4)		9 (20.5)		0.164
Urinary tract infection	2 (9.1)		1 (2.3)		0.531
Soft tissue skin infection	0 (0)		1 (2.3)		1.000
Sinusitis	4 (18.2)		2 (4.5)		0.173
Sepsis	3 (13.6)		3 (6.8)		0.650
Gastrointestinal disorder					
Nausea	12 (54.5)		33 (75.0)		0.093
Vomiting	8 (36.4)		25 (56.8)		0.117
Diarrhea	13 (59.1)		24 (54.5)		0.726
Bloating	4 (18.2)		5 (11.4)		0.704
Hepatic dysfunction					
Increase of γ-glutamyl transferase	20 (90.9)		29 (65.9)		**0.029**
Increase of lactate dehydrogenase	10 (45.5)		24 (54.5)		0.486
Increase of total serum bilirubin	8 (36.4)		20 (45.5)		0.481
Increase of alanine aminotransferase	8 (36.4)		20 (45.5)		0.481
Increase of aspartate aminotransferase	7 (31.8)		11 (25.0)		0.558
Increase of alkaline phosphatase	6 (27.3)		4 (9.1)		0.115
Renal insufficiency					
Increase of creatinine	2 (9.1)		1 (2.3)		0.531
Positive urine protein	8 (36.4)		15 (34.1)		0.855
Positive urine erythrocytes	4 (18.2)		16 (36.4)		0.130
Positive urine leukocytes	4 (18.2)		14 (31.8)		0.241
Others					
Mucositis	12 (54.6)		31 (70.5)		0.201
Intracranial hemorrhage	0 (0)		3 (6.8)		0.531
Pancreatitis	0 (0)		2 (4.5)		0.549
Epilepsy	0 (0)		3 (6.8)		0.531

NRM: non-relapse related mortality, aGVHD acute graft-versus-host disease, cGVHD chronic graft-versus-host disease, CMV cytomegalovirus, EBV Epstein-Barr virus

### Safety

Consistent with patients in the CON group, all patients in the Chi group manifested severe myelosuppression. The foremost non-hematologic adverse event (AE) observed was gastrointestinal tract reactions including diarrhea (Chi group, 59.1%; CON group, 54.9%; *P* = 0.724) and nausea (Chi group, 54.5%; CON group, 74.3%; *P* = 0.079). Hepatic dysfunction was more often observed in the Chi group. 20 patients evinced an elevation in γ-glutamyltransferase, as compared to the mBuCy group (90.9 vs. 65.9%, respectively; *P* = 0.029). (Table 2).

Seventeen (77.3%) patients administered the Chi regimen developed treatment-related infections, primarily pneumonia (54.6%). Severe adverse events including sepsis were reported in 3 cases (13.6%) of the Chi group and 3 cases (6.8%) of the mBuCy group (*P* = 0.650) (Table 2). All three cases in the Chi group were gram-negative infections. No instances of treatment-related death were reported in the Chi group during the treatment period.

### Non-relapse related mortality and overall survival

**Fig. 1:**
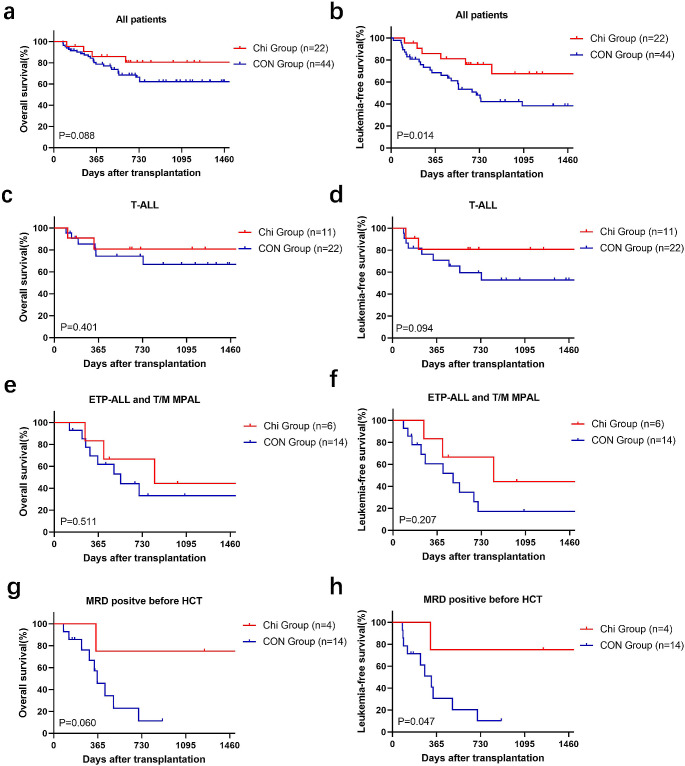
Kaplan-Meier curves of overall survival and leukemia-free survival. **(a)** Overall survival and **(b)** leukemia-free survival of all patients in the Chi group (*n* = 22) and CON group (*n* = 44). **(c)** Overall survival and **(d)** leukemia-free survival of T-ALL patients in the Chi group (*n* = 11) and CON group (*n* = 22). **(e)** Overall survival and **(f)** leukemia-free survival of ETP-ALL and T/ M MPAL patients in the Chi group (*n* = 6) and CON group (*n* = 14). **(g)** Overall survival and **(h)** leukemia-free survival of patients with MRD positive before HSCT in the Chi group (*n* = 4) and CON group (*n* = 14)

**Fig. 2:**
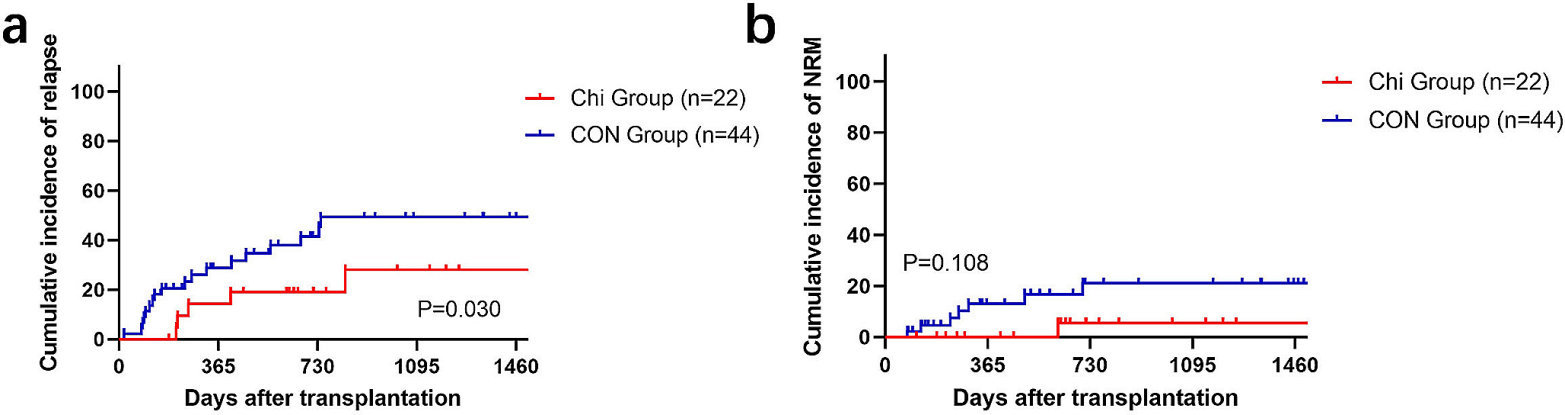
Cumulative incidence of relapse and non-relapse mortality patients. Cumulative of (**a**) relapse and (**b**) non-relapse mortality of all patients in the Chi group (*n* = 22) and CON group (*n* = 44)

The median follow-up time in the Chi group was 733 (range 368–1255) days, while that of the CON group was 684 (range 246–1219) days. There was no significant difference in OS between two groups (80.5% for Chi group vs. 60.4% for CON group, *P* = 0.063, Fig. 1a), while median OS was not reached in either group. The incidence of NRM was similar between the Chi and CON groups (5.5 vs. 21.1%, *P* = 0.108, Fig. 2b). The 2-year OS for T-ALL patients was similar in both groups (80.8 vs. 66.9%, *P* = 0.401, Fig. 1c). ETP-ALL and MPAL had similar biological characteristics, immunophenotypes, and poor outcomes [24, 25]. In our study, they were incorporated into the same group. And no differences were observed in two groups for patients with ETP-ALL and MPAL (66.7 vs. 33.2%, *P* = 0.511, Fig. 1e). Despite being not statistically significant, patients with MRD positive before HSCT in Chi group exhibited an advantage in 2-year OS (75.0 vs. 11.4%, *P* = 0.060). The univariate analysis of OS was shown in Table 3. In multivariate analysis, chidamide combined with mBuCy showed no correlation with OS for all patients (HR 0.34, 95%CI, 0.11–1.07; *P* = 0.064), while grade III-IV aGVHD (HR 4.72, 95%CI, 1.53–14.59; *P* = 0.007), MRD positive before HSCT (HR 3.22, 95%CI, 1.23–8.44; *P* = 0.018), and non-CR status of primary disease (HR 6.30, 95%CI, 1.96–20.31; *P* = 0.002) was associated with a lower OS (Table 4).

**Table 3:** Univariate analysis

Variable	HR (95%CI)	To LFS *P* value	HR (95%CI)	To OS *P* value
Conditioning regimen (Chi + mBuCy vs. mBuCy)	0.34 (0.14,0.83)	**0.018**	0.30 (0.10,0.91)	**0.033**
Diagnosis				
T-ALL	1		1	
T-LBL	0.85 (0.30,2.39)	0.760	1.20 (0.40,3.60)	0.743
ETP-ALL	2.16 (0.92,5.07)	**0.079**	1.76 (0.62,4.95)	0.287
T/M MPAL	1.57 (0.51,4.84)	0.433	1.69 (0.45,6.27)	0.435
Gender (Female vs. male)	1.59 (0.55,4.62)	0.391	0.90 (0.21,3.86)	0.884
Age > 35 years (Yes vs. No)	1.07 (0.50,2.30)	0.854	0.97 (0.40,2.39)	0.951
WBC > 100 × 10^9^/L> (Yes vs. No)	0.55 (0.21,1.48)	0.238	1.04 (0.39,2.75)	0.944
Complex karyotype (Yes vs. No)	1.36 (0.52,3.57)	0.529	1.37 (0.46,4.05)	0.572
Status of primary disease				
CR1	·1		1	
≥CR2	2.66 (1.04,6.80)	**0.041**	4.32 (1.46,12.73)	0.008
non-CR	4.90 (1.91,12.60)	**0.001**	8.67 (3.12,24.12)	**< 0.001**
MRD positive before HSCT (Yes vs. No)	2.20 (1.05,4.62)	**0.038**	2.89 (1.26,6.64)	**0.013**
Donor type				
haplo	1		1	
SIB	0.99 (0.30,3.31)	0.989	2.00 (0.67,6.02)	0.216
URD	0.49 (0.15,1.65)	0.248	0.43 (0.09,1.95)	0.271
Donor-recipient gender match (Yes vs. No)	1.17 (0.64,2.14)	0.621	0.95 (0.48,1.86)	0.885
Stem cell source (PBSC + BM vs. PBSC)	0.38 (0.12,1.16)	**0.088**	0.42 (0.12,1.53)	0.189
II-IV aGVHD (Yes vs. No)	1.80 (0.83,3.91)	0.138	2.85 (1.18,6.91)	**0.020**
III-IV aGVHD (Yes vs. No)	3.39 (1.42,8.09)	**0.006**	5.61 (2.09,15.06)	**0.001**
cGVHD (Yes vs. No)	1.03 (0.49,2.14)	0.947	0.83 (0.35,2.00)	0.679

T-ALL T-cell acute lymphoblastic leukemia, T-LBL T-cell acute lymphoblastic lymphoma, ETP-ALL early T-cell precursor acute lymphoblastic leukemia, T/M MPAL T/myeloid mixed phenotype acute leukemia, HSCT hematopoietic stem cell transplantation, CR complete remission, MRD minimal residual disease, Haplo Haploidentical donors, SIB HLA-matched sibling donors, URD HLA-matched unrelated donors, PBSC peripheral blood stem cells, BM bone marrow, aGVHD acute graft-versus-host disease, cGVHD chronic graft-versus-host disease

**Table 4:** Multivariate analysis

Variable	HR (95%CI)	To LFS *P* value	HR (95%CI)	To OS *P* value
Conditioning regimen (Chi + mBuCy vs. mBuCy)	0.23 (0.08,0.63)	**0.004**	0.34 (0.11,1.07)	**0.064**
Diagnosis				
T-ALL	1			
T-LBL	1.04 (0.30,3.56)	0.953		
ETP-ALL	1.66 (0.51,5.44)	0.405		
T/M MPAL	1.70 (0.48,6.07)	0.414		
Status of primary disease				
CR1	1		1	
≥CR2	3.51 (0.98,12.58)	0.053	2.73 (0.83,8.97)	0.098
non-CR	3.72 (1.09,12.73)	**0.036**	6.30 (1.96,20.31)	**0.002**
MRD positive before HSCT (Yes vs. No)	1.21 (0.46, 3.20)	0.694	3.22 (1.23,8.44)	**0.018**
Stem cell source (PBSC + BM vs. PBSC)	0.18 (0.04,0.76)	**0.020**		
III-IV aGVHD (Yes vs. no)	3.41(1.08,10.83)	**0.037**	4.72 (1.53,14.59)	**0.007**

CR complete remission, MRD minimal residual disease, Haplo Haploidentical donors, SIB HLA-matched sibling donors, URD HLA-matched unrelated donors, PBSC peripheral blood stem cells, BM bone marrow, aGVHD acute graft-versus-host disease, cGVHD chronic graft-versus-host disease

### Relapse and leukemia-free survival

At the time of the last follow-up, 5 patients in the Chi group and 20 patients in the CON group relapsed after transplantation. The median time from transplantation to relapse was 255 days and 266 days, respectively. The Chi group had a significantly lower 2-year CIR than the CON group (19.0 vs. 41.4%, *P* = 0.030) (Fig .2a). Furthermore, compared to the CON group, the Chi group had a better 2-year LFS (76.1 vs. 45.3%, *P* = 0.014, Fig. 1b). For patients with T-ALL, 2-year LFS was similar between Chi and CON group (80.8 vs. 59.5%, *P* = 0.094, Fig. 1d), while no difference was found between two groups for patients with ETP-ALL or T/M MPAL (66.7 vs. 17.3%, *P* = 0.207, Fig. 1f). In the subgroup of patients with MRD-positive before transplantation, the Chi group exhibited a better 2-year LFS (75.0 vs. 10.2%, *P* = 0.048). The univariate analysis of LFS was shown in Table 3. In multivariate analysis, chidamide combined with mBuCy (HR 0.23; 95%CI, 0.08–0.63; *P* = 0.004), and stem cells source of bone marrow stem cells combined with peripheral blood stem cells (PBSC + BM) (HR 0.18, 95%CI, 0.04–0.76; *P* = 0.020) showed a significant association with a higher LFS, while grade III-IV aGVHD (HR 3.41, 95%CI, 1.08–10.83; *P* = 0.037) and non-CR status of primary disease (HR 3.72, 95%CI, 1.09–12.73; *P* = 0.036) was associated with a lower LFS (Table 4).

## Discussion

Despite HSCT being a reliable treatment option, T-ALL/LBL is often accompanied by a dismal prognosis, with a high likelihood of post-transplant relapse. In our propensity matched cohort study, the combination of chidamide and mBuCy conditioning regimen improves the survival of patients without increasing the incidence of transplant-related mortality.

The possible mechanisms for chidamide to improve the mBuCy regimen efficacy may include: (1) Chidamide has a direct anti-leukemic effect: chidamide can block the cell cycle at the G0/G1 phase and induce apoptosis by activating the endogenous apoptotic signaling pathways simultaneously [26]. (2) Chidamide has a synergistic effect in combination with chemotherapy, especially the alkylating agent busulfan: preclinical studies had found that HDAC inhibitors lead to a more relaxed chromatin structure through chromatin remodeling, particularly hyperacetylation of lysine residues in the histone tails, which facilitates the expression of tumor suppressor genes as well as the action of chemotherapy [27–30]. (3) The poor prognosis of DT-negative T-ALL patients was associated with abnormal DNA aggregation leading to drug resistance (e.g. VP16, MTX) [31, 32], and chidamide may be able to overcome conventional chemotherapy resistance based on its chromatin-releasing activity. (4) Chidamide combined with chemotherapy regimens can effectively decrease MRD in T-ALL patients with *NOTCH1* mutation [20].

For T-ALL/LBL, especially high risk, a large portion of post-transplant relapse is due to failure to effectively eliminate MRD in bone marrow or extramedullary disease. Intensified conditioning regimens are expected to improve this situation. Our study confirmed that adding chidamide to the mBuCy regimen significantly attenuated the CIR without increasing the incidence of NRM. As a result, the patients who received the chi-intensified regimen achieved a better 2-year LFS compared to those who received the mBuCy conditioning regimen. Multivariate analysis also suggested that the Chi regimen was associated with superior LFS. Currently, TBI-based regimens are more recommended than chemo-based regimens for conditioning of T-ALL [33, 34]. The TBI-based conditioning regimens in ALL appears to be more effective than the mBuCy regimen, especially for T-ALL patients aged < 35 years can benefit significantly from the regimen of TBI based regimen (5-year LFS of 50% for TBI vs. 18% for chemo-only regimen or Bu-Cy regimens) [34–37]. However, in the haplo-HSCT setting, the application of the mBuCy regimen is more widespread and well established in our center. Recent RCT studies have also shown that, for ALL patients undergoing haplo-HSCT, there were no differences in 2-year OS, CIR, NRM, regimen-related toxicity, GVHD, or late effects between two regimens, indicating noninferiority of BuCy [38]. Given that chidamide has a synergistic effect in combination with the alkylating agent busulfan [27, 29, 30], this may enhance the efficacy of conditioning. Therefore, we choose to add chidamide to the mBuCy regimen rather than TBI-based regimens. Compared to the TBI-based conditioning regimen, a report from the acute leukemia working party of EBMT, our outcomes displayed a higher 2-year OS (80.8 vs. about 60%). Compared with the conventional chemo-based regimen (iv Bu-Cy and oral Bu-Cy), the survival difference was more significant (80.8 vs. about 30%) [34]. TBI-based regimens have advantages for the clearance of extramedullary disease, and in our study, two patients who still with extramedullary disease before transplantation both experienced extramedullary relapse and died after transplantation. There were four patients with MRD positive before transplantation in the Chi group and all were diagnosed with T-ALL, one patient died of relapse 329 days after transplantation, and the remaining three patients continued in leukemia-free survival. Compared to the mBuCy group, the MRD-positive patients in the Chi group exhibited a better LFS and an advantage OS (75.0 vs. 10.2%, 75.0 vs. 11.4%, respectively), which is consistent with previous research [20]. Hence, from the preliminary favorable results, T-ALL/LBL patients without extramedullary infiltration before transplantation may benefit from this chi-intensified regimen, especially for those with positive MRD. Unfortunately, for ETP-ALL and T/M MPAL, compared to a previous study in our center, no significant difference was seen in the OS and LFS [24]. Three patients with ETP-ALL or MPAL experienced relapse in the Chi group. One patient with MPAL died soon after relapse, and two patients with ETP-ALL prolonged survival with reinduction therapy and radiotherapy. As a high-risk subtype in T-ALL/LBL [2], ETP-ALL does not seem to benefit from the Chi-intensified conditioning regimen.

Two previous research have shown that chidamide had little toxicities in the autologous stem cell transplantation of non-Hodgkin’s lymphomas (chidamide 30 mg/day orally on days − 7, -4, 0, + 3, and chidamide 30 mg/day orally on days − 7, -4, + 1, +3) [21, 39]. In our study (chidamide 15 mg/day orally on days − 12, -9, -7, -5 for haploidentical donors and chidamide 15 mg/day orally on days − 11, -9, -7, -5 for HLA match siblings or unrelated donors), despite the Chi group being associated with a higher incidence of elevation in γ-glutamyltransferase, it did not result in fatal or severe toxicity when compared to the mBuCy regimen, and liver dysfunction all had been resolved after symptomatic treatment. Considering SAEs after transplantation were comparable between the two groups, we hold that this regimen is relatively safe and manageable.

It is acknowledged that there are some limitations in our study. Our evaluation was based on a retrospective analysis of a smaller sample size. Additionally, patient selection bias may have arisen during the enrollment. We also recognized that the cytotoxicity and efficacy of chidamide are influenced by the dosage regimen (timing, frequency, and dose), necessitating further optimization. To confirm our results, we anticipate conducting future prospective randomized controlled trials on a larger scale.

To our knowledge, this is the first study to combine chidamide into a conditioning regimen for allo-HSCT in T-ALL/LBL, and the preliminary results are encouraging. Our study suggests that compared with standard mBuCy regimens, chidamide combined with mBuCy conditioning regimen may be an effective and acceptable safety option.

### Electronic supplementary material

Below is the link to the electronic supplementary material.

Supplementary Material 1

## Data Availability

No datasets were generated or analysed during the current study.

## Abbreviations

T-ALL/LBL: T acute lymphoblastic leukemia/lymphoma
ETP-ALL: Early T-cell precursor acute lymphoblastic leukemia
T/M MPAL: T/myeloid mixed phenotype acute leukemia
Allo-HSCT: Allogeneic hematopoietic stem cell transplantation
MRD: Minimal residual disease
Haplo: Haploidentical
SIB: HLA-matched sibling
URD: HLA-matched unrelated
PBSC: Peripheral blood stem cells
BM: Bone marrow
GVHD: Graft-versus-host disease
aGVHD: Acute graft-versus-host disease
cGVHD: Chronic graft-versus-host disease
OS: Overall survival
LFS: Leukemia-free survival
CR: Complete remission
NR: No remission
PR: Partial remission
NRM: Non-relapse-related mortality
HDAC: Histone deacetylases
HDACi: Histone deacetylases inhibitor
CMV: Cytomegalovirus
EBV: Epstein-Barr virus
