# Supplementary material for: Chidamide combined with a modified Bu-Cy conditioning regimen improves survival in patients with T-cell acute lymphoblastic leukemia/lymphoma undergoing allogeneic hematopoietic stem cell transplantation
Source: Ann Hematol. 2024 Jun 20;103(8):3083–93. doi: 10.1007/s00277-024-05849-y (PMC11283404; doi:10.1007/s00277-024-05849-y)
Supplement: Supplementary file 1 — Supplementary Material 1 [file 277_2024_5849_MOESM1_ESM.docx]

**Table S1: Baseline covariates before and after propensity score matching.**

| Variables | Level | Before Matching | | | After Matching | | |
| --- | --- | --- | --- | --- | --- | --- | --- |
|  |  | CON group | Chi group | SMD^△^ | CON group | Chi group | SMD^△^ |
| n |  | 82 | 22 |  | 44 | 22 |  |
| Age (mean (SD)) |  | 28.20 (10.56) | 27.91 (10.76) | -0.027 | 29.18 (11.58) | 27.91 (10.76) | -0.118 |
| Diagnosis (%) | T-ALL | 43 (52.4) | 11 (50.0) | -0.049 | 22 (50.0) | 11 (50.0) | 0.000 |
|  | T-LBL | 18 (22.0) | 5 (22.7) | 0.019 | 8 (18.2) | 5 (22.7) | 0.108 |
|  | ETP-ALL | 12 (14.6) | 4 (18.2) | 0.092 | 9 (20.5) | 4 (18.2) | -0.059 |
|  | T/M MPAL | 9 (11.0) | 2 (9.1) | -0.066 | 5 (11.4) | 2 (9.1) | -0.079 |
| Gender (%) | Male | 60 (73.2) | 19 (86.4) | 0.384 | 40 (90.9) | 19 (86.4) | -0.132 |
|  | Female | 22 (26.8) | 3 (13.6) | -0.384 | 4 (9.1) | 3 (13.6) | 0.132 |
| Status of disease before HSCT (%) | CR1 | 64 (78.0) | 17 (77.3) | -0.019 | 34 (77.3) | 17 (77.3) | 0.000 |
|  | ≥CR2 | 10 (12.2) | 3 (13.6) | 0.042 | 6 (13.6) | 3 (13.6) | 0.000 |
|  | Non-CR | 8 (9.8) | 2 (9.1) | -0.023 | 4 (9.1) | 2 (9.1) | 0.000 |
| Donor type (%) | Haplo | 64 (78.0) | 17 (77.3) | -0.019 | 33 (75.0) | 17 (77.3) | 0.054 |
|  | SIB | 7 (8.5) | 3 (13.6) | 0.149 | 5 (11.4) | 3 (13.6) | 0.066 |
|  | URD | 11 (13.4) | 2 (9.1) | -0.150 | 6 (13.6) | 2 (9.1) | -0.158 |
| Stem cell source (%) | PBSC | 59 (72.0) | 19 (86.4) | 0.420 | 35 (79.5) | 19 (86.4) | 0.199 |
|  | PBSC+BM | 23 (28.0) | 3 (13.6) | -0.420 | 9 (20.5) | 3 (13.6) | -0.199 |
| Donor-recipient gender match (%) | Male-male | 50 (61.0) | 13 (59.1) | -0.038 | 29 (65.9) | 13 (59.1) | -0.139 |
|  | Male-female | 19 (23.2) | 3 (13.6) | -0.278 | 4 (9.1) | 3 (13.6) | 0.132 |
|  | Female-male | 9 (11.0) | 3 (13.6) | 0.078 | 7 (15.9) | 3 (13.6) | -0.066 |
|  | Female-female | 4 (4.9) | 3 (13.6) | 0.255 | 4 (9.1) | 3 (13.6) | 0.132 |
| Relapsed or refractory (%) | NO | 58 (70.7) | 14 (63.6) | -0.147 | 30 (68.2) | 14 (63.6) | -0.094 |
|  | YES | 24 (29.3) | 8 (36.4) | 0.147 | 14 (31.8) | 8 (36.4) | 0.094 |

SMD Standardized Mean Difference, T-ALL T acute lymphoblastic leukemia, T-LBL T acute lymphoblastic lymphoma, ETP-ALL early T-cell precursor acute lymphoblastic leukemia, T/M MPAL T/myeloid mixed phenotype acute leukemia, HSCT hematopoietic stem cell transplantation, CR complete remission, Haplo Haploidentical donors, SIB HLA-matched sibling donors, URD HLA-matched unrelated donors, PBSC peripheral blood stem cells, BM bone marrow
